# A Neurophysiological Approach for Evaluating Noise-Induced Sleep Disturbance: Calculating the Time Constant of the Dynamic Characteristics in the Brainstem

**DOI:** 10.3390/ijerph13040369

**Published:** 2016-03-25

**Authors:** Junta Tagusari, Toshihito Matsui

**Affiliations:** 1 Graduate School of Engineering, Kyoto University, Kyoto daigaku-katsura Nishikyo-ku, Kyoto 615-8530, Japan; junta.tagusari@gmail.com; 2 Graduate School of Engineering, Hokkaido University, Kita 13 Nishi 8 Kita-ku, Sapporo 060-8628, Japan

**Keywords:** sleep disturbance, neurophysiology, brainstem, Phillips–Robinson model, time constant

## Abstract

Chronic sleep disturbance induced by traffic noise is considered to cause environmental sleep disorder, which increases the risk of cardiovascular disease, stroke, diabetes and other stress-related diseases. However, noise indices for the evaluation of sleep disturbance are not based on the neurophysiological process of awakening regulated by the brainstem. In this study, through the neurophysiological approach, we attempted (1) to investigate the thresholds of awakening due to external stimuli in the brainstem; (2) to evaluate the dynamic characteristics in the brainstem and (3) to verify the validity of existing noise indices. Using the mathematical Phillips–Robinson model, we obtained thresholds of awakening in the brainstem for different durations of external stimuli. The analysis revealed that the brainstem seemed insensitive to short stimuli and that the response to external stimuli in the brainstem could be approximated by a first-order lag system with a time constant of 10–100 s. These results suggest that the brainstem did not integrate sound energy as external stimuli, but neuroelectrical signals from auditory nerve. To understand the awakening risk accumulated in the brainstem, we introduced a new concept of “awakening potential” instead of sound energy.

## 1. Introduction

### 1.1. Indices for Night-Time Noise

Noise-induced sleep disturbance is a serious environmental problem that is associated with health concerns, such as environmental sleep disorder and risk of cardiovascular disease [1,2,3,4,5,6,7,8]. The estimated number of noise-induced behavioral awakenings per year due to commercial aircraft would be 133 in the worst case at the indoor night-time equivalent level ($L_{\mathrm{night},i}$) of 40 dB [2]. The HYENA study (“Hypertension and Exposure to Noise near Airports”) [3] focused on night-time noise around the airport revealed a significant association for risk of hypertension with night-time equivalent level ($L_{\mathrm{night}}$), but not day-time equivalent level ($L_{\mathrm{day}}$). The WHO Regional Office for Europe considered health implications, such as cardiovascular disease, stroke, diabetes and other stress-related diseases, due to night-time noise and developed a guideline: $L_{\mathrm{night}}$ of 40 dB. [4]. The World Health Organization (WHO) Regional Office for Europe [5] estimated the disability-adjusted life years (DALYs) lost from self-reported sleep disturbance to be 903,000 years for the 285 million population living in agglomerations with >50,000 inhabitants. Moreover, recent epidemiological studies reported associations with stroke, diabetes and obesity, which are also caused by sleep disorder [8].

Noise-induced awakenings in response to noise events have been studied both in the field and laboratory settings [9,10,11,12,13,14,15]. To obtain the relationship between the probability of awakening and sound levels, noise indices, such as sound exposure level (*SEL*), maximum sound level using a “fast” time constant ($L_{\mathrm{Amax},\mathrm{fast}}$) and $L_{\mathrm{night}}$, were used in these studies. A review of field and laboratory studies [9] suggests that the *SEL* is more highly correlated to the probability of behavioral awakening than the $L_{\mathrm{Amax},\mathrm{fast}}$, which has lead to the more widespread use of *SEL* for measurements of night-time noise [10,11,12,13,15]. In addition, the *SEL* is calculated by integrating sound energy, which is similar to the $L_{\mathrm{night}}$, which is a widely-used index for the evaluation of the long-term effects of night-time noise exposure; therefore, the evaluation using *SEL* was useful to set the night-time noise guideline using the $L_{\mathrm{night}}$ [2,4]. However, the prediction using the *SEL* fails to account for appreciable amounts of variance in dose-response relationships of awakening and is not freely generalizable from airport to airport [16]. Fidell *et al.* [16] reported that standard deviations of the *SEL* were more closely related to the probability of behavioral awakening than absolute sound levels; however, it still awaits proof of its value [8].

The calculation of integrating sound energy was introduced because of practical reasons, but not based on physiological determinants of the human auditory system or the physiology of sleep and wakefulness in the brain. In the human auditory system, neuro-electrical signals generated by noise stimuli are relayed to the brain or the brainstem for processing [17], though the relevance of sound energy to the integration processes affecting the sleep-wake switch is unclear. On the other hand, the neurophysiology of sleep and wakefulness has been increasingly recognized by recent studies [18,19]. A neurophysiological approach is considered to be essential for evaluating night-time noise and assessing the usefulness of existing noise indices.

### 1.2. Neurophysiological View of Wakefulness and Sleep

Neurophysiologically, states of sleep and wakefulness in the forebrain are controlled by nuclei in the brainstem and hypothalamus that are referred to as the ascending arousal system (AAS) and the ventrolateral preoptic nucleus (VLPO), respectively [18,19]. The AAS can be divided into two groups of nuclei: the monoaminergic (MA) group, which is active during wakefulness, and the acetylcholine-related (ACh) group, which is active during both wakefulness and rapid eye movement (REM) sleep. During wakefulness, activation of the AAS and particularly the MA nuclei cause the suppression of activity in the VLPO. Conversely, during non-REM sleep, the VLPO suppresses activity in the MA. In addition, orexinergic neurons (Orx) in the lateral hypothalamus function to activate the MA group and prolong wakefulness [20]. Sleep-wake dynamics can therefore be characterized by the mutually exclusive inhibition of the MA and VLPO. This system facilitates extended periods of sleep or wakefulness and rapid transitions between the two states that are akin to a “flip-flop”-type circuit [18]. The transitions between the states would be in the order of minutes [21]. The interaction between the MA and ACh groups is also known to control the transition between REM and non-REM sleep states and to regulate the 90-min ultradian rhythm [22,23].

Circadian and homeostatic sleep drive inputs are transmitted to the VLPO in order to generate a periodic oscillation of sleep and wakefulness. Cues from the biological clock in the suprachiasmatic nucleus (SCN) allow synchronization of the sleep-wake cycle with circadian rhythm [19,24]. The homeostatic drive similarly promotes sleep via a yet-unidentified structure or possibly the accumulation of an endogenous substance during wakefulness that signals the need for sleep [19,23].

Numerous mathematical models have been developed to represent the activation of brainstem nuclei during sleep and wakefulness, such as the two-process model [25,26,27] and the mutual inhibition model [28,29,30,31,32]. These models have been validated against existing biological evidence and have provided profound insights into the dynamics of sleep and wakefulness. The two-process model explains the sleep-wake cycle as a function of homeostatic and circadian processes that increase sleep pressure and modulate the threshold of the transition between wakefulness and sleep, respectively. This model has been widely used for the evaluation of sleep-wakefulness cycle; however, the rapid transition of the two states is difficult to evaluate using this model [32]. The mutual inhibition model was subsequently developed based on the physiological observation of mutually exclusive activation and interaction between the MA nuclei in the AAS and the VLPO nuclei. This model includes both the circadian sleep-wakefulness cycle and rapid transition of the two states, which enables one to replicate the “flip-flop”-type circuit [28,29,30,32].

The mathematical Phillips–Robinson model [28,29] is a mutual inhibition model, wherein a relatively small number of equations and parameters determine the mathematical behavior of the model based on neurophysiological findings. Although this model does not account for the effect of sleep stages, including REM sleep, this model has provided significant insights into the process of awakening elicited by external stimuli [29,33,34], the effects of caffeine [35] and chronotype [36]. There are several studies focusing one the relationship between sleep and external stimuli using this model where external drives were introduced to the MA (impulsive external stimuli, including noise events [29,33] and “wake effort” to maintain the system in a wakeful state [34]) and the VLPO (light signal to the photoreceptors in the eye [37]). Fulcher *et al.* [31] studied narcolepsy in a modification of the Phillips–Robinson model that incorporated the influence of Orx. In particular, the implementation of the Phillips–Robinson model by Fulcher *et al.* [33] provided useful insights into the process of awakening in response to an individual noise event: the authors examined thresholds of awakening that were defined using the time to return to a stable sleep state. Importantly, the return to a stable sleep state fluctuates with circadian and homeostatic drive inputs to the VLPO. By converting electrical stimuli into auditory tones, Fulcher and colleagues were able to replicate variation in awakening thresholds during sleep. This study is a testament to the usefulness of the Phillips–Robinson model in representing and studying the reaction of the brainstem to external stimuli.

Of note, variation in the voltage changes of the VLPO and MA in response to different durations of stimuli were also illustrated in the study by Fulcher *et al.* [33]; however, the effect of stimulus duration on the awakening threshold was not fully discussed. Since individual noise events occurring repeatedly during sleep have specific durations, the awakening response of the brainstem to different durations of external stimuli is of interest to the predictions of noise-induced sleep disturbance.

In summary, recent advances in our understanding of the neurophysiological dynamics of sleep in the brainstem have yielded useful mathematical models that provide not only accurate representations of brainstem sleep-wake dynamics, but also the opportunity to generate novel insights into sleep-wake neurophysiology. The mathematical Phillips–Robinson model enables the evaluation of brainstem responses to external stimuli, and several studies have employed this model to evaluate awakening in response to external stimuli, including noise events [29,33]. However, these studies did not take into consideration the neurophysiological relevance of the noise indices used. Furthermore, no studies to date have evaluated awakening responses to different durations of external auditory stimuli. A grasp of the relative abilities of different durations of noise events to induce awakening and the duration of the resulting awakening effect is vital for understanding noise-induced sleep disturbance.

In the present study, we first investigated variations in awakening thresholds in response to different durations of external stimuli using the mathematical Phillips–Robinson model. Continuous (<1000 s) and constant external stimuli were assumed to derive the relationship between the awakening threshold and the duration of the stimulus. In addition, we applied a first-order lag system to external stimuli with different time constants in order to evaluate the integration system of the brainstem. We converted electrical thresholds to sound levels and examined the validity of current noise indices for a single noise event using existing experimental results. The relationship between average awakening levels and the duration of auditory stimuli was used to evaluate the validity of the existing noise indices, $L_{\mathrm{Amax},\mathrm{fast}}$ and *SEL*.

## 2. Methods

### 2.1. Phillips–Robinson Model

**Figure 1:**
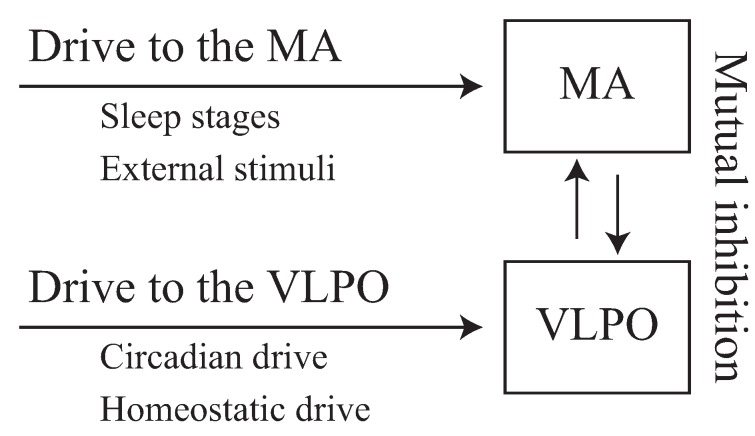
Illustration of the schematic diagram of the Phillips–Robinson model. The monoaminergic (MA) and ventrolateral preoptic nucleus (VLPO) nuclei are activated by external drives that influence sleep stages, external stimuli, as well as internal circadian and homeostatic drives. Mutual inhibition between the MA and VLPO comprises the sleep-wake switch.

In this study, we used the mathematical Phillips–Robinson model [28,29] of sleep and wakefulness that describes the activity of populations of neurons in the brainstem and enables the quantitative evaluation of the sleep-wake switch when a brief awakening is triggered by an external stimulus [33]. A schematic diagram of this model is shown in Figure 1. For neurophysiological validity and simplicity, only the MA and VLPO nuclei were included in this model.

Each cell population has a mean cell body potential relative to the resting potential, $V_{j}\left(t\right)\;\left(\mathrm{mV}\right)$, and a mean firing rate, $Q_{j}\left(t\right)\;\left(s^{-1}\right)$, where *j* takes the character of *m* representing the MA or *v* representing the VLPO. The relationship of $Q_{j}$ to $V_{j}$ is approximated by the sigmoid function of:

(1) $$Q_{j}=S\left(V_{j}\right)=\frac{Q_{\max}}{1+\exp\left[-\left(V_{j}-\theta \right)/\sigma^{\prime }\right]}$$

where $Q_{\max}\;\left(s^{-1}\right)$ is the maximum possible firing rate, θ(mV) is the mean firing threshold relative to the resting potential and $\sigma^{\prime }\pi /\sqrt{3}\;\left(\mathrm{mV}\right)$ is its standard deviation. Differential equations of $V_{j}\left(t\right)$ are given by:

(2) $$\left\{\begin{array}{ccc} \tau_{v}{\dot{V}}_{v}+V_{v} & = & \nu_{vm}Q_{m}+D_{v}\\ \tau_{m}{\dot{V}}_{m}+V_{m} & = & \nu_{mv}Q_{v}+D_{m} \end{array}\right.$$

where $\nu_{jk}$ weights the input from populations *k* to *j*, $\tau_{j}\;\left(s\right)$ is the decay time of the neuromodulator expressed by group *j* and $D_{j}\;\left(\mathrm{mV}\right)$ represents an external drive to population *j*. The external drives are given in Equation (3). $D_{m}$ includes a component of the external stimulus, and $D_{v}$ changes in response to circadian and homeostatic drives, such that:

(3) $$\left\{\begin{array}{ccc} D_{m} & = & A+D_{\mathrm{ext}}\\ D_{v} & = & \nu_{vc}C+\nu_{vh}H \end{array}\right.$$

where, A(mV) is a constant value related to sleep stages and the wakefulness-stabilizing drive of the Orx, $D_{\mathrm{ext}}\;\left(\mathrm{mV}\right)$ is a drive related to external stimuli, *C* is the circadian drive and H(nMs) is the somnogen level of the homeostatic drive. The variable *C* is calculated by:

(4) $$C=\sin\omega t+c_{0}$$

and *H* is calculated by:

(5) $$\chi \dot{H}+H=\mu Q_{m}$$

where $\omega \;(h^{-1})=2\pi /24$, $c_{0}$ and μ(nMs) are constants and χ(s) is the characteristic time for somnogen clearance. In the calculation of this model, we applied the same values of the constant parameters as presented in the existing studies (see Table 1 in Fulcher *et. al.* [33] or Table 1 in Phillips *et. al.* [29]).

**Figure 2:**
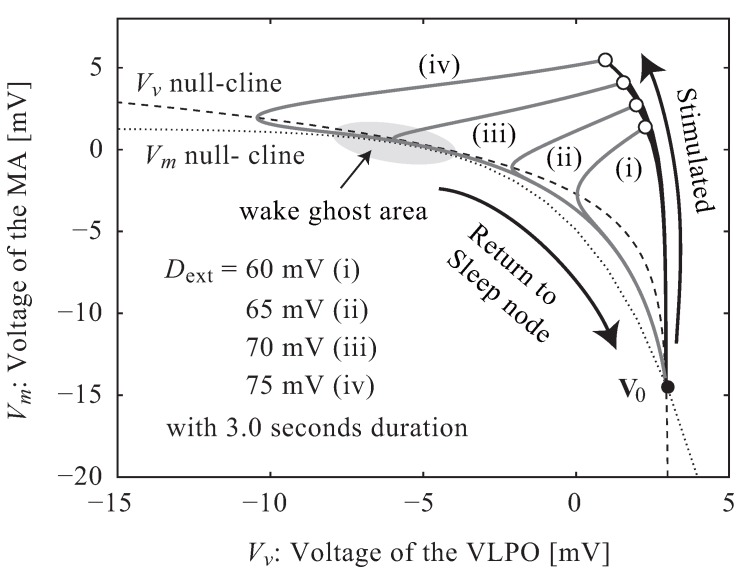
The behavior of the potential, V(t), in response to external stimuli. The nullclines of $V_{v}$ and $V_{m}$ and their intersection, $V_{0}$, were obtained from Equation (2). V(t) moves upward in response to a stimulus (thick black line) before returning to $V_{0}$ (grey line).

This model allows for the calculation of a neuro-electrical threshold of awakening due to external stimuli. Figure 2 shows the behavior of the potentials, $V\left(t\right)=(V_{v}\left(t\right),V_{m}\left(t\right))$, in $V_{v}$- $V_{m}$ coordinates. The nullclines of $V_{v}$ and $V_{m}$ were obtained by substituting zeros for $\dot{V_{v}}$ and $\dot{V_{m}}$ in Equation (2). The intersection of these curves, $V_{0}$, is a stable node representing sleep where V(t) remains constant, even if time progresses, and returns to this point even after V(t) is temporarily disturbed by an external stimulus presented during sleep. There is an area called the “wake ghost” where the nullclines are close, but do not intersect; in this area, the potentials, V(t), move slowly. As a result, after V(t) moves upward in response to perturbation by an external stimulus (thick black line), an extended period of time is needed before the potential can return to the sleep node, and accordingly, it takes much time to return to the sleep node (grey line).

**Figure 3:**
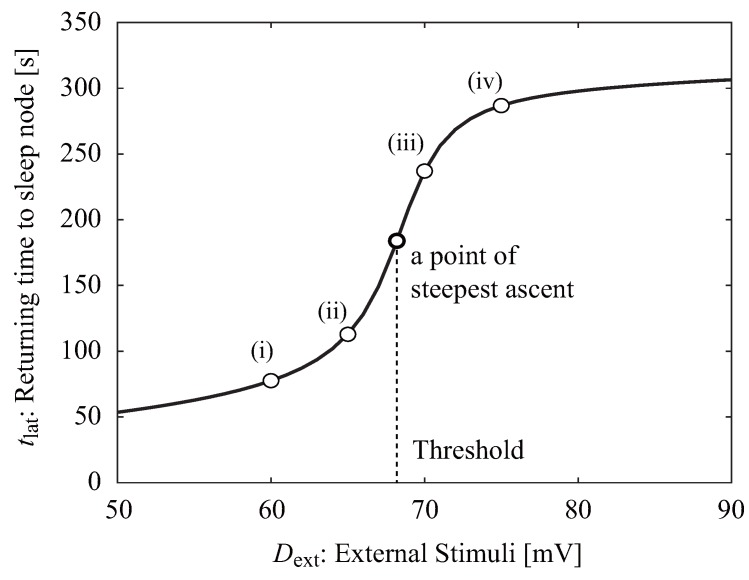
Relationship between the time to return to the sleep node, $t_{\mathrm{lat}}$, and the strength of the external stimulus, $D_{\mathrm{ext}}$. The neuroelectrical threshold of awakening, $D_{\mathrm{ext}}^{*}$, is defined as the point at which the return time ascent is the steepest.

The time required to return to the sleep node, $t_{\mathrm{lat}}$, is dependent on the voltage of the external stimulus, $D_{\mathrm{ext}}$ (Figure 3). The numbering in this figure (i to iv) correspond to that in Figure 2. If $D_{\mathrm{ext}}$ exceeds a certain value, the return time increases remarkably because V(t) returns to $V_{0}$ through the “wake ghost” area. The neuroelectrical threshold of awakening, $D_{\mathrm{ext}}^{*}$, is defined as the external stimulus corresponding to the point of the steepest ascent.

### 2.2. Neuroelectrical Threshold of Awakening

External stimuli, $D_{\mathrm{ext}}$, input to the MA were assumed to be constant ($D_{c}\;\left(\mathrm{mV}\right)$) and to have a duration of $t_{d}\;\left(s\right)$ that is relatively short (<1000 s) compared to the circadian rhythm:

(6) $$D_{\mathrm{ext}}=\left\{\begin{array}{ccc} D_{c} & \mathrm{for} & 0\leq t\leq t_{d}\\ 0 & \mathrm{for} & t_{d}<t \end{array}\right.$$

Neuro-electrical thresholds of awakening were obtained as a function of $t_{d}$. The effects of circadian rhythm input into the VLPO and the input of the wakefulness-stabilizing drive into the MA on thresholds were evaluated by substituting empirical values for $D_{v}$ and *A*, respectively. $D_{v}$ fluctuates with the duration of sleep, and the fluctuation of *A* was left out in the Phillips–Robinson model, while the fluctuation is characterized by Orx [31].

The brainstem response was approximated using a first-order time lag system integrating the fluctuations of the external stimuli, where the maximum output value, $\bar{D_{\max}}$, represents the neuroelectrical threshold at each time constant, $t_{c}$, expressed by:

(7) $$\bar{D}\left(t_{0}\right)=\int_{-\infty }^{t_{0}}e^{-(t_{0}-t)/\tau }D\left(t\right)\;dt$$

The relationship between the neuroelectrical thresholds of awakening and the duration of external stimuli allowed calculation of the system’s time constant. We set the time constant to 0.1, 1.0, 10, 30 and 100 s.

We also varied the time constant of the $\tau_{m}$ and $\tau_{v}$. A nominal value of 10 s was selected based on the transition time between sleep and wakefulness, since the true value represents a complex neurophysiological effect and is therefore difficult to obtain [28]. However, their value can shift the thresholds of awakening [33].

### 2.3. Conversion of Neuroelectrical Thresholds into Sound Level

To validate the $L_{\mathrm{Amax},\mathrm{fast}}$ and *SEL* indices from a neurophysiological viewpoint, these indices were generated from neuroelectrical values using experimentally-obtained values reported by Bonnet *et al.* [38]. In the original study, the authors broadcast 3 s-long 1000-Hz pure tones and measured the amplitude (dB) required to wake subjects (6 male) from Sleep Stage 2. Though sleep state was monitored by EEG, awakening was defined as a button press and verbal response from the subject, which corresponded to “behavioral awakening” as measured in several noise studies [39]. We used experimental results from the placebo nights in the Bonnet study to calculate the average awakening level. Table 1 shows the average awakening levels in five time blocks from the original study where the $D_{v}$ calculated from elapsed time from sleep onset and the neuroelectrical threshold of awakening was dependent on the calculated $D_{v}$.

The following criteria were imposed to define awakening: at least 5 min of well-defined Sleep Stage 2, at least 30 min of continuous sleep and at least 10 min without body movement or muscle artifact greater than 6 seconds. However, the first awakening made after initial Sleep Stage 2 did not meet the criteria. Furthermore, this awakening level of 30.8 dB is lower than 35 dB, the sound level threshold reported for EEG awakening [4]. A time period of 5 min seemed too short to evaluate the awakening effects of auditory stimuli and was therefore excluded from conversions of neuroelectrical stimuli into sound levels.

We used the following functions for converting neuroelectrical stimuli into sound levels:

(8) $$\begin{array}{cccccccc} & \mathrm{Linear}\;(i) & : & & D_{c} & =c_{1}\left(L_{A}-c_{2}\right) & & \mathrm{for}\;L_{A}\geq c_{2} \end{array}$$

(9) $$\begin{array}{cccccccc} & \mathrm{Linear}\;(\mathrm{ii}) & : & & D_{c} & =c_{3}\left(L_{A}-35\right) & & \mathrm{for}\;L_{A}\geq 35\;\mathrm{dB} \end{array}$$

(10) $$\begin{array}{cccccccc} & \mathrm{Power} & : & & D_{c} & =c_{4}{\left(L_{A}-35\right)}^{c_{5}} & & \mathrm{for}\;L_{A}\geq 35\;\mathrm{dB} \end{array}$$

(11) $$\begin{array}{cccccccc} & \mathrm{Exponential} & : & & D_{c} & =10^{(L_{A}-c_{6})/10}+c_{7} & & \end{array}$$

where $D_{c}$ is the strength of external stimulus, $L_{A}$ is the A-weighted sound level and $c_{1}$ to $c_{7}$ are constants that were obtained by applying the least-square method to the results of Table 1 (excluding the awakening level of 5 min after falling asleep). In Equations (9) and (10), an awakening threshold level of 35 dB was used because the threshold of awakening was so estimated based on previous laboratory and field studies [4]. The exponential function used in Equation (11) is for the conversion of neuroelectrical stimuli into sound power. If neuroelectrical stimuli depend on sound power, this function fits well into the experimental results.

Next, we examined the relationship between average awakening levels (for $L_{\mathrm{Amax},\mathrm{fast}}$ and *SEL*) and the duration of noise events, $t_{d}$, using the neuroelectrical thresholds of awakening and the corresponding sound level values. An ideal noise index gives a constant awakening level that does not vary with the duration of the noise events; however, average awakening levels using actual noise indices would likely vary in this manner. Therefore, we examined the validity of the existing noise indices based on the variation of the average awakening levels.

## 3. Results

### 3.1. Neuroelectrical Threshold of Awakening

**Figure 4:**
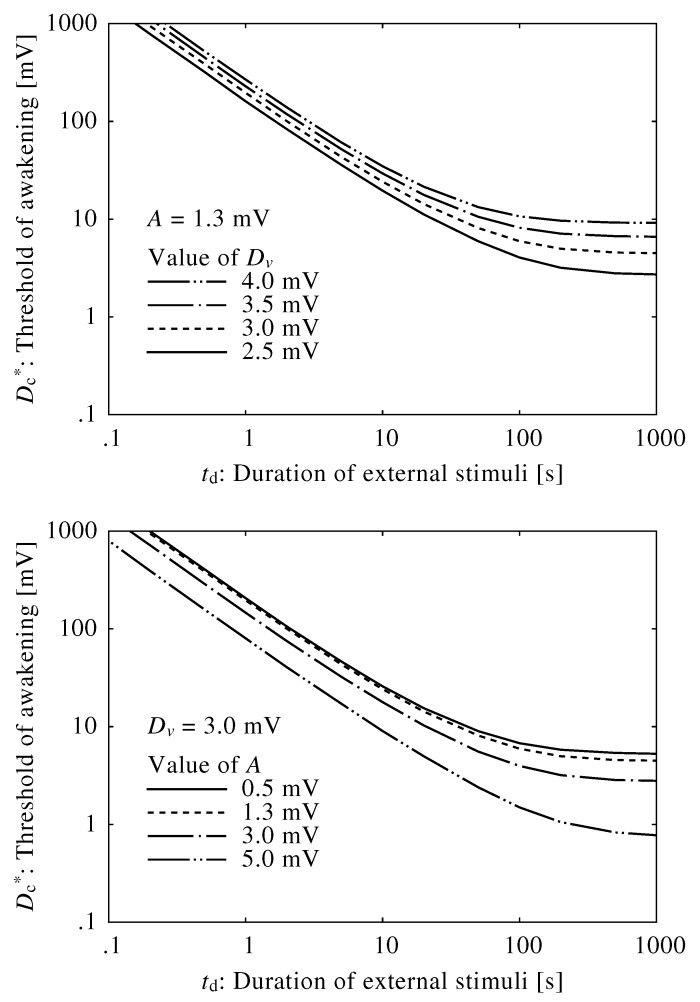
Relationship between neuroelectrical thresholds of awakening in response to external stimuli and varied duration of external stimuli using different values of $D_{v}$ and *A*.

Figure 4 shows the relationships between the neuroelectrical thresholds of awakening and the duration of the external stimuli, $t_{d}$. The brainstem model was insensitive to short external stimuli, and the thresholds were extremely high. Alternatively, the thresholds for sufficiently long external stimuli were constant, indicating that stimuli below the threshold would not cause an awakening regardless of their duration or consistency. The threshold curves changed very little when using different values of *A* and $D_{v}$.

**Figure 5:**
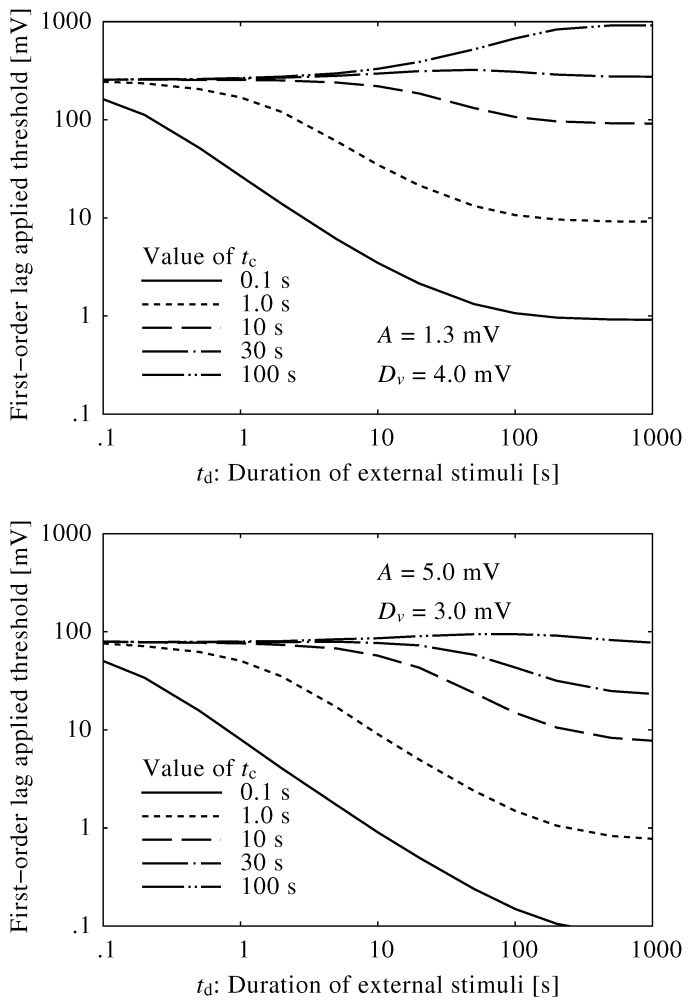
Relationship between neuroelectrical thresholds of awakening in response to external stimuli and varied duration of external stimuli, where a first-order lag system was applied to the thresholds (see Equation (7)) using different values of the time constant, $t_{c}$.

Figure 5 shows the relationships between the duration of the external stimuli and the $\bar{D_{\max}}$, given five different time constants, $t_{c}$, where the values of *A* and $D_{v}$ vary. When $t_{c}=$ 10–100 s, the $\bar{D_{\max}}$ remained almost constant, suggesting that the brainstem model reaction time can be approximated using a time lag system with a time constant in this range. It is important to note that these values are fundamentally different from both the “fast” (0.125 s) and “slow” (1.0 s) time-weighting typically used to integrate sound energy in studies on the effects of community noise, including night-time noise that might cause sleep disturbance.

**Figure 6:**
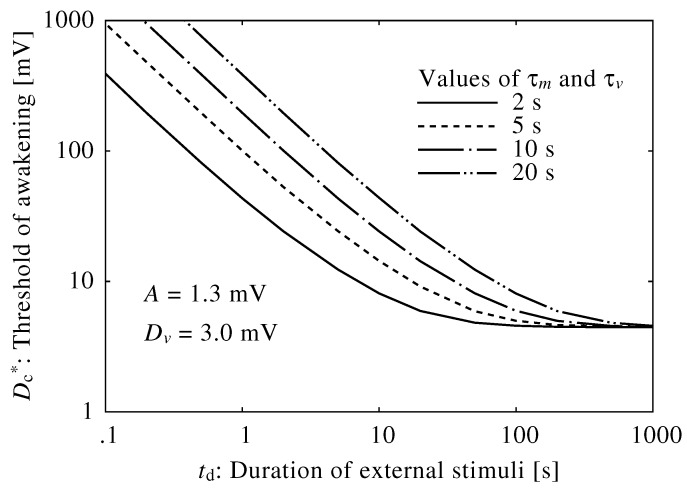
Relationship between neuroelectrical thresholds of awakening in response to external stimuli and the duration of the external stimuli using different values of $\tau_{m}$ and $\tau_{v}$.

The time constant in the brainstem model primarily depended on the decay time of the neuromodulators, $\tau_{v}$ and $\tau_{m}$, which were set to 10 s. Figure 6 shows neuroelectrical thresholds of awakening calculated using different time constants, $\tau_{m}$ and $\tau_{v}$. Fluctuations in decay time shifted neuroelectrical thresholds of awakening; specifically, thresholds of awakening increased as the decay time increased. However, the parameters of decay time represent complex neurophysiological effects, and true decay time is difficult to obtain. They were set based on the transition time between sleep and wakefulness.

### 3.2. Conversion into Sound Level

**Figure 7:**
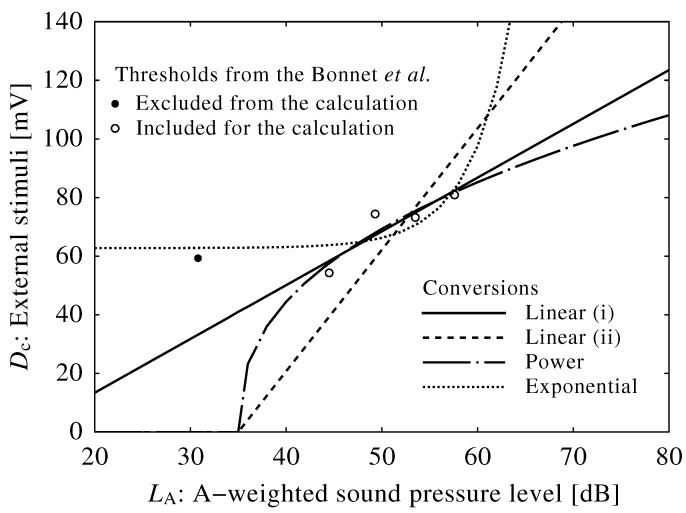
Experimental and mathematical results (see Table 1, white and black circles) and conversion curves of neuroelectrical stimuli to sound pressure levels. Note that the threshold falling after five minutes (black circle) was excluded from the calculation for obtaining conversions.

We obtained four conversion curves as follows (see also Figure 7):

(12) $$\begin{array}{ccccccc} & \mathrm{Linear}\;(i) & : & & D_{c}= & 1.84(L_{A}-12.7) & \mathrm{for}\;L_{A}\geq 12.7\;\mathrm{dB}, \end{array}$$

(13) $$\begin{array}{ccccccc} & \mathrm{Linear}\;(\mathrm{ii}) & : & & D_{c}= & 4.14(L_{A}-35) & \mathrm{for}\;L_{A}\geq 35\;\mathrm{dB}, \end{array}$$

(14) $$\begin{array}{ccccccc} & \mathrm{Power} & : & & D_{c}= & 23.2{\left(L_{A}-35\right)}^{0.405} & \mathrm{for}\;L_{A}\geq 35\;\mathrm{dB}, \end{array}$$

(15) $$\begin{array}{cccccccc} & \mathrm{Exponential} & : & & D_{c}= & 10^{(L_{A}-44.6)/10}+62.8 & & . \end{array}$$

A conversion curve of Equation (12) was in agreement with reported experimental results. However, it yielded an unexpectedly low threshold sound level of 12.7 dB compared to the empirical threshold of 35 dB used in Equations (13) and (14). Conversion curves of Equations (13) and (14) were also in agreement with the results, although there was a wide range of extrapolation. A fourth curve of Equation (15) was not in agreement with the reported experimental results, and its adoption would have required the constant input of external stimuli to the brainstem. Thus, Equation (15) was deemed unsuitable for conversion, and this suggests that the input of external stimuli to the brainstem model is not associated with sound power.

**Figure 8:**
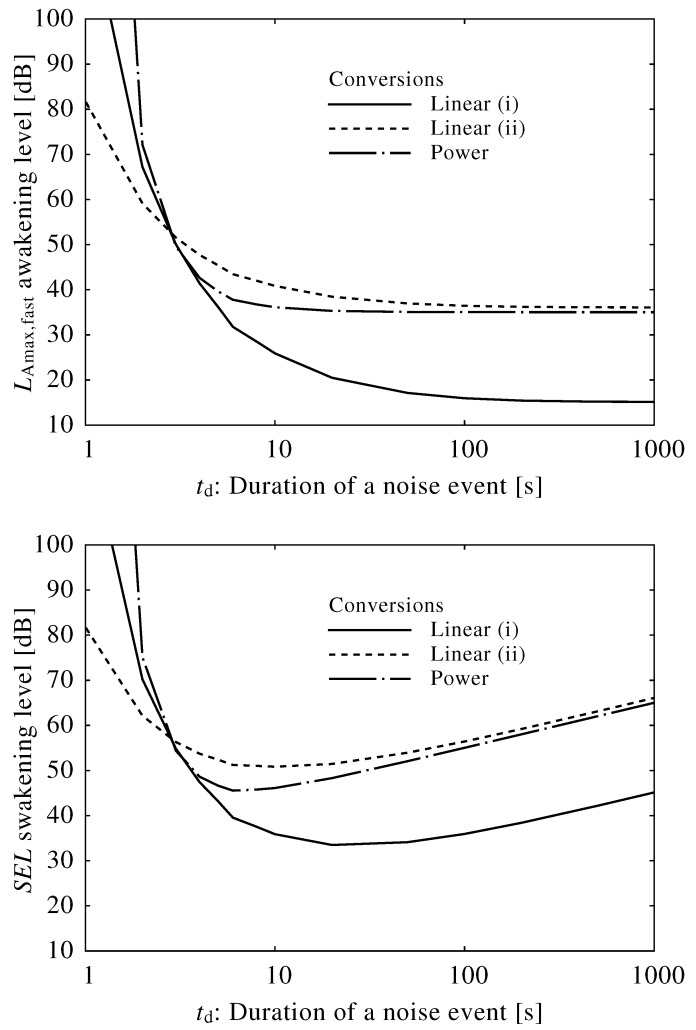
Average awakening levels using $L_{\mathrm{Amax},\mathrm{fast}}$ and sound exposure level (*SEL*), where A=1.3 (mV) and $D_{v}=3.0$ (mV). Equations (12) through (14) were used to convert neuroelectrical thresholds into sound levels.

We used three conversion equations (excluding Equation (15)) to obtain average awakening levels using $L_{\mathrm{Amax},\mathrm{fast}}$ and *SEL*, as shown in Figure 8, where A=1.3 (mV) and $D_{v}=3.0$ (mV). Similar trends were obtained using alternative values for *A* and $D_{v}$.

For both the $L_{\mathrm{Amax},\mathrm{fast}}$ and *SEL*, awakening levels due to short duration noises were extremely high, suggesting that short duration noises are unlikely to induce awakening and disrupt sleep based on our model. Furthermore, this finding shows that the $L_{\mathrm{Amax},\mathrm{fast}}$ and *SEL* overestimate the risk of awakening in response to short duration noises. The steep slopes plotted in these graphs are likely the result of the biological integration system: external stimuli are input to the brainstem and then integrated as a function of sound level over a period of 10–100 s.

Given the number of studies that link $L_{\mathrm{Amax},\mathrm{fast}}$ and *SEL* to sleep disturbance, these indices may provide sufficient estimates of awakening risk in certain circumstances. For instance, in our study, the $L_{\mathrm{Amax},\mathrm{fast}}$ values were fairly constant regardless of the duration of the noise event, indicating that this index is appropriate for longer duration noise events. However, this index should not be used for short duration noise events, since the awakening level abruptly increases when the duration falls below 10 s. Conversely, the *SEL* yielded a lower minimum awakening level and a higher level than that of the $L_{\mathrm{Amax},\mathrm{fast}}$, though the awakening level increased with the duration of noise, indicating that the *SEL* is not useful for long duration noise events. Table 2 shows the ranges for which the $L_{\max}$ and *SEL* may be useful in the evaluation of single noise events under the conditions described in Figure 8. Within the specified ranges, awakening levels do not fluctuate more than 3 dB from the minimum value. The valid ranges for noise indices are preliminary, and Table 2 only indicates a qualitative trend.

## 4. Discussion

The aims of this study are (1) to investigate the thresholds of awakening in the brainstem due to different durations of external stimuli, (2) to evaluate the dynamic characteristics in the brainstem and (3) to verify the validity of the existing noise indices of $L_{\mathrm{Amax},\mathrm{fast}}$ and *SEL*.

First, we evaluated the relationship between neuroelectrical thresholds of awakening and the duration of external stimuli using the mathematical Phillips–Robinson model. Neuroelectrical thresholds were inversely proportional to the duration for short external stimuli, while the thresholds were constant for long stimuli. This finding suggests that the brainstem integrates external stimuli over a certain time constant. Therefore, we applied a first-order lag system to the response in the brainstem, and found the time constant of 10–100 s in the lag system of the brainstem. These values are considerably longer than those used in traditional “fast” or “slow” sound weightings, suggesting that the existing noise indices may not accurately model the brainstem’s integration system.

The relationship between neuroelectrical thresholds and external stimuli was also affected by the $D_{v}$ related to circadian rhythm and the $D_{m}$ related to sleep stages and the wakefulness-stabilizing drive from Orx. However, the threshold curve changed very little by these values, which suggests that the time constant of the lag system would not be changed by these values during sleep. In contrast, the duration of the external stimulus necessary for awakening correlates with the parameters, $\tau_{m}$ and $\tau_{v}$, of the Phillips–Robinson model, and changes in $\tau_{m}$ and $\tau_{v}$ accordingly shift the threshold curve. Thus, $\tau_{m}$ and $\tau_{v}$ have a considerable impact on the time constant in the brainstem’s integration of external stimuli. However, the values of $\tau_{m}$ and $\tau_{v}$ were determined empirically using the transition time between sleep and wakefulness, and no reasonable explanation could be found to use a value other than the empirical value of 10 s. The parameters of mutual inhibition models [28,30,31,32] are set to longer values as the parameters to adjust for the rapid transition between sleep and wakefulness (“flip-flop”-type circuit) [21], which underpins the obtained time constant of 10–100 s in the brainstem.

Based on the findings in this study, longer durations of noise events are more likely to induce awakenings because of the time constant of the brainstem, which is consistent with the results reported by Passchier-Vermeer *et al.* [40], where the probability of motility reaction in response to a longer duration of noise events was 1.5-times higher than the average duration. Lercher *et al.* [41] also reported that the longer duration of noise events contributes to higher sleep medication intake.

We should note that, as mentioned in the previous studies [29,33], neuroelectrical thresholds changed according to the $D_{v}$ related to circadian rhythm. The threshold and $D_{v}$ are relatively small in the early phases of sleep and increase over time until they reach a peak around midnight and subsequently decrease. Accordingly, the smallest values for the threshold and $D_{v}$ are observed in the early morning. Although the effect of sleep stage is excluded in the Phillips–Robinson model, our results indicated that noise-induced sleep disturbances are most likely to occur in the early morning, as has been noted elsewhere [14,15].

We tried to convert the neuroelectrical thresholds of awakening into average awakening levels in $L_{\mathrm{Amax},\mathrm{fast}}$ and *SEL* based on existing experimental results [38]. Although very limited data were available, we assumed three conversion equations. Each average awakening level changes substantially with fluctuations in the duration of the external stimulus, which suggests that both the $L_{A,\max}$ and *SEL* overestimate the risk of awakening due to a short noise and that the *SEL* overestimates the risk due to a long noise.

The difference of the dose-response relationships between sound levels and the probability of awakenings in different sound sources might be partly because of the overestimation and underestimation of awakening risk using existing noise indices. For instance, Passchier-Vermeer *et al.* [2] reported that the short duration of noise events due to military aircraft is more likely to induce behavioral awakenings than commercial aircraft at the same *SEL*, which suggests that the *SEL* would overestimate the risk of awakening due to commercial aircraft (or underestimate military aircraft).

To understand the risk of awakening accumulated in the brainstem, we introduce a concept of “awakening potential” for the evaluation of night-time noise. Awakening potential is a neuroelectrical potential of awakening from the auditory nerve, which would be integrated in the brainstem with a time constant of 10–100 s. We [42] have developed an index to evaluate awakening risk based on the integration of “awakening potential” according to the results obtained in this paper. Further studies based on neurophysiology and epidemiology would be necessarily to confirm and modify the validity of the developed index.

## 5. Conclusions

In this study, we investigated the dynamics of the brainstem that regulates sleep and wakefulness using the mathematical Phillips–Robinson model. The calculation with the model indicated that the response in the brainstem would be approximated by a first-order lag system integrating external stimuli with a time constant of roughly 10–100 s. In addition, we converted the neuroelectrical stimuli into average awakening levels using existing experimental results, which suggests that both the $L_{\mathrm{Amax},\mathrm{fast}}$ and *SEL* would overestimate awakening in response to a short noise and that the *SEL* would overestimate a long noise. We instead propose the use of the “awakening potential”, an accumulating risk of wakefulness in the brainstem, as a noise index for the evaluation of noise-induced sleep disturbance.

## Figures and Tables

**Table 1:** Average awakening levels in Bonnet *et al.* [38] and neuroelectrical thresholds calculated from the Phillips–Robinson model. Values represent the average results from the six study subjects.

Results from Bonnet *et al.*		Results from the Phillips–Robinson model	
Time after falling asleep (min)	Average awakening levels (dB)	$D_{v}$: Drive to the VLPO (mV)	$D_{c}^{*}$: Neuroelectrical threshold (mV)
5	30.8	2.62	59.3
110	49.3	3.24	74.4
220	57.6	3.50	80.9
330	53.5	3.19	73.2
420	44.5	2.46	54.3

**Table 2:** Ranges over which the *SEL* or $L_{\mathrm{Amax},\mathrm{fast}}$ values do not fluctuate more than 3 dB.

Conversion Equation	$t_{d}$: Range of the Noise Duration (s)	
	*SEL*	$L_{\mathrm{Amax},\mathrm{fast}}$
Linear (i)	$10\leq t_{d}\leq 100$	$50\leq t_{d}$
Linear (ii)	$4\leq t_{d}\leq 50$	$20\leq t_{d}$
Power	$4\leq t_{d}\leq 20$	$6\leq t_{d}$

## References

[1] Babisch W. (2002). The noise/stress concept, risk assessment and research needs. Noise Health.

[2] Passchier-Vermeer W. (2003). Night-Time Noise Events and Awakening.

[3] Jarup L., Babisch W., Houthuijs D., Pershagen G., Katsouyanni K., Cadum E., Dudley M., Savigny P., Seiffert I., Swart W. (2008). Hypertension and exposure to noise near airports: The HYENA study. Environ. Health Perspect..

[4] World Health Organization Regional Office for Europe (2009). Night Noise Guidelines for Europe.

[5] World Health Organization Regional Office for Europe (2011). Burden of Disease from Environmental Noise. Quantification of Healthy Life Years Lost in Europe.

[6] Babisch W. (2014). Updated exposure-response relationship between road traffic noise and coronary heart diseases: A meta-analysis. Noise Health.

[7] Münzel T., Gori T., Babisch W., Basner M. (2014). Cardiovascular effects of environmental noise exposure. Eur. Heart J..

[8] Basner M., Brink M., Bristow A., de Kluizenaar Y., Finegold L., Hong J., Janssen S., Klaeboe R., Leroux T., Liebl A. (2015). ICBEN review of research on the biological effects of noise 2011-2014. Noise Health.

[9] Pearsons K.S., Barber D.S., Tabachnick B.G., Fidell S. (1995). Predicting noise-induced sleep disturbance. J. Acoust. Soc. Am..

[10] Fidell S., Pearsons K., Tabachnick B., Howe R., Silvati L., Barber D.S. (1995). Field study of noise-induced sleep disturbance. J. Acoust. Soc. Am..

[11] Fidell S., Howe R.R., Tabachnick B.G., Pearsons K.S., Sneddon M.D. (1995). Noise-Induced Sleep Disturbance in Residences Near Two Civil Airports.

[12] Fidell S., Howe R., Tabachnick B., Pearsons K., Silvati L., Sneddon M., Fletcher E. (1998). Field Studies of Habituation to Change in Nighttime Aircraft Noise and of Sleep Motility Measurement Methods.

[13] Passchier-Vermeer W., Vos H., Steenbekkers J., van der Ploeg F., Groothuisoudshoorn K. (2002). Sleep Disturbance and Aircraft Noise Exposure.

[14] Basner M., Samel A., Isermann U. (2006). Aircraft noise effects on sleep: Application of the results of a large polysomnographic field study. J. Acoust. Soc. Am..

[15] Anderson G., Miller N. (2007). Alternative analysis of sleep-awakening data. Noise Control Eng. J..

[16] Fidell S., Tabachnick B., Mestre V., Fidell L. (2013). Aircraft noise-induced awakenings are more reasonably predicted from relative than from absolute sound exposure levels. J. Acoust. Soc. Am..

[17] Rees A., Palmer A.R. (2010). Level and spectrum. The Oxford Handbook of AUDITORY SCIENCE: The Auditory Brain.

[18] Saper C.B., Chou T.C., Scammell T.E. (2001). The sleep switch: Hypothalamic control of sleep and wakefulness. Trends Neurosci..

[19] Saper C.B., Scammell T.E., Lu J. (2005). Hypothalamic regulation of sleep and circadian rhythms. Nature.

[20] Saper C.B., Fuller P.M., Pedersen N.P., Lu J., Scammell T.E. (2010). Sleep state switching. Neuron.

[21] Merica H., Fortune R.D. (2004). State transitions between wake and sleep, and within the ultradian cycle, with focus on the link to neuronal activity. Sleep Med. Rev..

[22] Gallopin T., Fort P., Eggermann E., Cauli B., Luppi P.H., Rossier J., Audinat E., Muhlethaler M., Serafin M. (2000). Identification of sleep-promoting neurons in vitro. Nature.

[23] Strecker R.E., Morairty S., Thakkar M.M., Porkka-Heiskanen T., Basheer R., Dauphin L.J., Rainnie D.G., Portas C.M., Greene R.W., McCarley R.W. (2000). Adenosinergic modulation of basal forebrain and preoptic/anterior hypothalamic neuronal activity in the control of behavioral state. Behav. Brain Res..

[24] Pace-Schott E., Hobson J. (2002). The neurobiology of sleep: genetics, cellular physiology and subcortical networks. Nat. Rev. Neurosci..

[25] Borb A.A., Achermann P. (1999). Sleep homeostasis and models of sleep regulation. J. Biol. Rhythms.

[26] Achermann P., Borbély A.A. (2003). Mathematical models of sleep regulation. Front. Biosci..

[27] Achermann P. (2004). The Two-Process Model of Sleep Regulation Revisited. Aviat. Space Environ. Med..

[28] Phillips A., Robinson P. (2007). A quantitative model of sleep-wake dynamics based on the physiology of the brainstem ascending arousal system. J. Biol. Rhythms.

[29] Phillips A., Robinson P. (2008). Sleep deprivation in a quantitative physiologically based model of the ascending arousal system. J. Theor. Biol..

[30] Rempe M.J., Best J., Terman D. (2009). A mathematical model of the sleep/wake cycle. J. Math. Biol..

[31] Fulcher B.D., Phillips A.J.K., Postnova S., Robinson P.A. (2014). A Physiologically Based Model of Orexinergic Stabilization of Sleep and Wake. PLoS ONE.

[32] Skeldon A.C., Dijk D.J., Derks G. (2014). Mathematical Models for Sleep-Wake Dynamics: Comparison of the Two-Process Model and a Mutual Inhibition Neuronal Model. PLoS ONE.

[33] Fulcher B.D., Phillips A.J.K., Robinson P.A. (2008). Modeling the impact of impulsive stimuli on sleep-wake dynamics. Phys. Rev. E Stati. Nonlinear Soft Matter Physics.

[34] Fulcher B., Phillips A., Robinson P. (2010). Quantitative physiologically based modeling of subjective fatigue during sleep deprivation. J. Theor. Biol..

[35] Puckeridge M., Fulcher B.D., Phillips A.J.K., Robinson P.A. (2011). Incorporation of caffeine into a quantitative model of fatigue and sleep. J. Theor. Biol..

[36] Phillips A., Chen P., Robinson P. (2010). Probing the mechanisms of chronotype using quantitative modeling. J. Biol. Rhythms.

[37] Skeldon A.C., Derks G., Dijk D.J. (2016). Modelling changes in sleep timing and duration across the lifespan: Changes in circadian rhythmicity or sleep homeostasis?. Sleep Med. Rev..

[38] Bonnet M., Webb W., Barnard G. (1979). Effect of flurazepam, pentobarbital, and caffeine on arousal threshold. Sleep.

[39] Basner M., Griefahn B., Berg M.V.D. (2010). Aircraft noise effects on sleep: Mechanisms, mitigation and research needs. Noise Health.

[40] Passchier-Vermeer W., Vos H., Janssen S.A., Miedema H.M. (2007). TNO Summary Report 2007-D-R0012/A Sleep and Traffic Noise Summary Report.

[41] Lercher P., Brink M., Rudisser J., Van Renterghem T., Botteldooren D., Baulac M., Defrance J. (2010). The effects of railway noise on sleep medication intake: Results from the ALPNAP-study. Noise Health.

[42] Tagusari J., Takashima T., Furukawa S., Matsui T. (2016). Night-Time Noise Index Based on the Integration of Awakening Potential. Int. J. Environ. Res. Public Health.

